# Nutritional Care in Cystic Fibrosis Across the Arab World: Regional Heterogeneity, Current Evidence, and Future Directions

**DOI:** 10.3390/nu18193187

**Published:** 2026-09-26

**Authors:** Ala K. Shaikhkhalil, Mutasim Abu-Hasan, Serena Sharpe, Fadi Asfour

**Affiliations:** 1 Division of Pediatric Gastroenterology, Hepatology and Nutrition, Sheikh Khalifa Medical City, Abu Dhabi P.O. Box 51900, United Arab Emirates; 2 Pulmonary Medicine, Sidra Medicine, Doha P.O. Box 26999, Qatar; 3 Clinical Nutrition and Allied Health, Sidra Medicine, Doha P.O. Box 26999, Qatar; 4 Department of Pediatrics, University of Utah, Salt Lake City, UT 84413, USA; 5 Primary Children’s Cystic Fibrosis Center, Intermountain Children’s Health, Salt Lake City, UT 84413, USA

**Keywords:** cystic fibrosis, nutrition, Arab world, Middle East and North Africa, malnutrition, growth, pancreatic insufficiency, pancreatic enzyme replacement therapy, CFTR modulators, health disparities

## Abstract

Background: cystic fibrosis (CF) is a genetic disease which was previously thought to predominantly affect people of European ancestry but has been increasingly diagnosed in other populations. Although respiratory disease is the principal manifestation, poor nutritional status is a central determinant of pulmonary outcomes and survival. There are gaps in the literature on epidemiology, genetics, clinical manifestations and nutritional outcomes of CF in the Arab world. Methods: in this narrative review, we aim to synthesize and analyze nutrition-relevant data and explore how these relate to epidemiology, diagnostic pathways, genotype-phenotype relationships, and availability and access to care. Results: the published evidence across the region is heterogenous in nature but shows that malnutrition in CF is dominant in reported pediatric cohorts. The overall poor nutritional outcomes of CF in the Arab world reflect the multifactorial effects of disease biology, diagnostics, healthcare capacity, and treatment access, as well as psychosocial and social and structural determinants of health. Conclusions: improving CF-related nutritional status in the region requires early diagnosis of the disease, ideally through newborn screening, proactive intervention, multidisciplinary care, access to therapies including modulator therapy, and the establishment of collaborative registries that capture existing genetic diversity and healthcare disparities while tracking clinical and nutritional outcomes.

## 1. Introduction

Cystic fibrosis (CF) is an autosomal recessive, life-shortening, multisystem disease caused by pathogenic variants in the cystic fibrosis transmembrane conductance regulator (CFTR) gene [1]. Impaired CFTR-mediated chloride and bicarbonate transport across the epithelium causes thick secretions and impaired innate immunity resulting in chronic airway disease, pancreatic insufficiency, gastrointestinal dysfunction, and other systemic complications [1]. Although CF was historically regarded as a disease affecting predominantly populations of European ancestry, it is now increasingly diagnosed in diverse populations worldwide [1,2].The true prevalence of CF in any region of the world remains unknown because of limited access to necessary diagnostic tools such as newborn screening, sweat chloride testing, and genetic analysis. Genetic testing is particularly important in detecting local CFTR variants [2,3,4,5,6].

Nutritional status in people with CF (PwCF) is closely linked to their pulmonary function, morbidity, and survival [7,8]. The etiology of malnutrition in PwCF is multifactorial, including increased energy expenditure, pancreatic insufficiency, gastrointestinal dysfunction, recurrent infections, and inadequate dietary intake [9,10].

Life expectancy in PwCF has improved in many high-income countries, even before the era of CFTR modulator therapy due to advances in advances in early diagnosis, access to specialized multidisciplinary care, and standardized treatment approaches, including proactive nutritional management [11,12].

In these settings, the nutritional phenotype of CF has also changed, particularly with the introduction of highly effective CFTR modulator therapy. Undernutrition has become less common, while overweight and obesity are now increasingly recognized [7,13]. This pattern, however, cannot be generalized globally. Unequal access to early diagnosis, standardized multidisciplinary care, essential nutritional therapies, and effective medications, including CFTR modulators likely contributes to malnutrition and poor outcome [14].

Arab countries share strong linguistic, historical, and cultural connections. However, they vary substantially in their population genetics, healthcare infrastructure, economic resources, political systems, and levels of social stability. Therefore, the Arab world provides valuable context for examining nutritional outcomes among PwCF who live in such diverse settings.

Published studies from several cohorts in Arab countries report delayed diagnosis, high rates of malnutrition, fat malabsorption, and severe respiratory disease [3,6]. In certain countries of the Arab world with more advanced healthcare systems and access to CFTR modulators, nutritional phenotypes may be changing, although regional evidence is very limited [13]. The available data suggest the existence of two nutritional realities occurring in parallel: persistent malnutrition in most of the published cohorts on the one hand, and signs of nutritional recovery in few select cohorts that receive CFTR modulators on the other.

A previously published review of CF in Arab countries cited delayed diagnosis, malnutrition, limited specialized care, and gaps in treatment as important contributors to poor outcomes [15]. Since that review, the regional literature has expanded and CF care has entered the modulator era in parts of the region, but malnutrition and poor clinical outcomes remain prominent in published cohorts. The relationship of this persistent malnutrition to pancreatic phenotype, treatment access, and healthcare and social conditions remains poorly characterized [3,6].

In this review, we synthesize available evidence, highlighting different regional patterns and identifying gaps in the current published evidence. We will focus on nutritional status and growth, in addition to prevalence and drivers of malnutrition including access to diagnosis and multidisciplinary care, and the emerging impact of CFTR modulators across the region.

## 2. Review Approach

This narrative review examined English-language literature relevant to CF nutrition and nutrition-related outcomes across the 22 Arab countries through 31 July 2026.

Because nutritional outcomes in CF need to be interpreted within the broader clinical and healthcare context, the literature search began broadly. PubMed and PubMed Central were searched using cystic fibrosis together with regional and individual country names to identify literature describing many aspects of CF across the Arab world including epidemiology, genetics and genotype-phenotype relationships, pancreatic function, newborn screening, data registries, and healthcare access. These broader searches established the regional clinical and healthcare context for the subsequent focus on CF nutrition.

Literature search then focused on studies reporting nutritional status, growth, malnutrition, undernutrition, micronutrient status, pancreatic phenotype, pancreatic enzyme replacement therapy (PERT), and nutritional interventions. Studies directly reporting nutritional or growth-related outcomes formed the core of the review. The broader CF literature provided clinical epidemiological, genetic, diagnostic, and healthcare context in the Arab Countries. Reference lists of primary studies and review articles were also examined to identify additional publications.

A.K.S conducted the literature search, extracted data and synthesized the findings. F.A. assisted with obtaining full-text publications when needed.

Because the available studies differed widely in their design, populations, treatment eras, and outcome definitions, we summarized the findings narratively. Single-patient case reports and non-English publications were excluded. Studies were not excluded based on their quality. However, methodological limitations of the cited studies were considered during presentation of the results and interpretation of the data.

Türkiye was included in the search as a regional comparator because of its geographic proximity and the relatively recent development of organized CF care in the country, including establishment of national newborn screening, registry-based data collection, and quality-improvement initiatives [16,17]. Türkiye, therefore, provided a more regionally relevant comparator as opposed to Western countries that have long-established CF care systems.

Terminology used in the original studies, such as, “failure to thrive” and “nutritional failure,” was retained when reporting findings. Otherwise, we use the more neutral terms of “growth faltering”, “undernutrition” and “malnutrition,” while recognizing that terminology and their psychosocial implications may vary across languages and clinical contexts.

## 3. The CF Landscape Across the Arab World

### 3.1. Epidemiology, Recognition, and Diagnostic Pathways

The true prevalence of CF across the Arab world remains unknown because epidemiologic studies are sparse and the existent ones derive prevalence estimates using different methods and populations. A systematic review published in 2021 identified 17 epidemiologic studies from 9 Arab countries, most of which were small hospital-based series, regional cohorts, or estimates extrapolated from limited screening or genetic data [6]. An update published in 2024 found 10 additional studies from 8 countries and concluded that progress in defining the prevalence of CF and its disease burden in the region remains modest [3].

The absence of region-wide data, however, should not be interpreted as evidence that CF is rare in the region, but more likely a reflection of the limited diagnostic and reporting capacity. Countries with stronger healthcare systems are more likely to diagnose patients with CF, maintain follow-up, and generate data than countries that remain poorly represented in the literature.

Two reports from the United Arab Emirates (UAE) demonstrate that delayed recognition and severe disease can persist even in a high-resource setting. In 1995, Dawson and Frossard described 12 Emirati children with CF who have marked growth impairment and severe multisystem disease at a time when CF was still considered uncommon locally [18]. Decades later, delayed diagnosis remained evident in an adult Emirati cohort of 39 PwCF: almost half had been diagnosed after 10 years of age, and nearly one-third after 16 years. Median Percent Predicted Forced Expiratory Volume in 1 s (ppFEV_1_) was 49.5%, compared to 71.1% in contemporary UK adult CF population. Chronic *Pseudomonas aeruginosa* colonization affected 32 patients (82%), which was nearly twice as high as the corresponding UK prevalence [19].

Saudi Arabia has contributed one of the largest published single-center cohorts in the region. Banjar et al. reviewed registry data for 430 PwCF followed at King Faisal Specialist Hospital and Research Centre between 1998 and 2018 [20]. The mean age at diagnosis was 3.46 years, and median survival was approximately 22 years. Consanguinity data was available in 242 patients, 201(83%) of these patients had consanguineous parents. Familial clustering was also common: 107 of 238 patients with available data (45%) had positive family history for CF. However, positive family history of CF was reported to help in the diagnosis of only 41 of the 430 patients (9.5%) [20].

Other informative regional data came from Oman and Qatar. In Oman, Fass et al. estimated the prevalence of CF un the population at approximately 1 in 8264 and reported greater clustering in the northeast [21]. A later multicenter study from the same country using different methodology identified a total of 227 patients and reported a prevalence estimate of 10.3 per 100,000 among Omani nationals younger than 30 years [22]. In Qatar, a historical review based on clinical and genetic criteria, identified 82 known patients and noted that no national registry was available at the time [23]. Even in these two countries with relatively better-described patient population and strong clinical and research settings, longitudinal data on growth and nutritional outcomes were limited.

Jordan has moved towards a more complete national data collection with the establishment of its first electronic CF registry, which has identified 385 patients [24]. Most of the patients were younger than 20 years and the mean age at diagnosis was approximately 3 years. Unfortunately, the registry excluded deceased patients and often key clinical data were often missing from the registry report, including sweat chloride results, genotyping, spirometry, and anthropometric measurements. The missing data limit the registry’s ability to describe the disease burden and guide future improvements in nutritional status and clinical care on a national level.

More recently, data from a single-center registry of 120 children followed at Ain Shams University in Egypt between 2022 and 2024 provided one of the larger contemporary pediatric CF cohorts reported from the region. In this cohort, failure to thrive was reported in 58.3% [25].

Other countries in the region published data that came from pediatric cohorts of various sizes. A single-center study in Syria reported 173 children with confirmed CF, with high consanguinity and substantial early mortality [26]. In Tunisia, 32 children were identified over 21- year period in a single referral center [27]. A subsequent Tunisian national reported 123 children with CF from twelve university departments, with a median age at diagnosis of 5 months and undernutrition rate of 55.2% [28]. In Algeria, a recent case series of 34 children reported frequent delayed diagnosis, chronic diarrhea, and growth retardation [29]. The first Sudanese report of CF consisted of 35 children, also described high rate of consanguinity and failure to thrive [30]. Morocco has very limited comparable published clinical cohort data. In one population-based genetic study of 150 healthy adults, two were found to be carriers of CFTR variant p.Phe508del which correspond to a carrier frequency of 1 in 75. Based on these finding, together with assumptions regarding the distribution of other CFTR variants and the rate of consanguinity in Morocco, the authors estimated a possible CF prevalence of approximately 1 in 1680 to 1 in 4150 [31]. These estimates were model-based and derived from carrier rate in a small sample of healthy adults, rather than from diagnosed cases or a national registry.

In summary, the available CF epidemiological data remain limited since most of the studies are hospital-based and are retrospective in nature. The available data do not provide reliable national estimates of prevalence, survival, clinical manifestations, or nutritional outcomes. These limitations, together with the absence of comprehensive national registries in much of the region, also constrain attempts to understand the regional CFTR variation and its relationship to the clinical and nutritional phenotypes.

### 3.2. Regional CFTR Variation and Clinical Implications

The available genetic literature indicates the presence of substantial CFTR variation across the Arab world, although the evidence is geographically uneven [4,5]. Al-Sadeq et al. identified 115 reported CFTR variants from 72 publications, 12 were recurrent, 85 were shared with non-Arab populations and 18 were considered potentially Arab-specific at the time of review [4]. Variant data were available from 18 out of the 22 Arab countries and showed that p.Phe508del was the most geographically widespread variant, but it was not the dominant variant in every country.

The Arab world does not have a genetically homogeneous population. Substantial ethnic and ancestral diversity exists within countries as well as between countries. Founder effects also play an important role in shaping local CFTR variants [32]. Therefore, findings from a single community or referral center cohort cannot be generalized or assumed to represent an entire country.

Data from the Gulf countries, for example, show how differently CFTR variants are distributed. Saudi Arabia provides one of the largest regional datasets with 48 distinct CFTR variants, six of them were novel and were identified in 396 PwCF from 312 families. No single variant accounted for more than 16% of alleles [33]. In Oman, variant p.Ser549Arg accounted for 75% of disease alleles in North Al Batinah region [21,22]. A recent Bahraini study identified 18 CF-causing variants in 56 PwCF, with p.Phe508del accounting for only 7.6% of the alleles [34]. Data from the UAE also showed significant variation within the same country: all 15 affected children with CF from 10 Emirati Bedouin families were homozygous for p.Ser549Arg. On the other hand, 6 children from 7 affected families of Baluch origin were homozygous for p.Phe508del [35].

Wide genotype variation has also been reported in the Levant countries. In Jordan, for example, genetic screening of 84 children and 66 parents identified 24 CFTR variants, with p.Phe508del accounting for only 7.4% [36]. In Iraq, a 34-variant panel genetic test was able to identify pathogenic variants in only a minority of clinically diagnosed patients [37]. A Palestinian study using next-generation sequencing identified 18 unique CFTR variants among 60 unrelated patients [38], In Lebanon, one study reported a diverse spectrum in which p.Phe508del and p.Asn1303Lys were among the most frequently detected variants [39].

North African studies showed similar wide genetic variations. In Tunisia, complete CFTR analysis in 68 unrelated patients identified 12 variants accounting for 90% of alleles; variant p.Phe508del was the most frequent at 47.1%, followed by p.Glu1104Ter at 16.2% [40]. In Algeria, genetic analysis of 36 children with CF has identified 15 CFTR variants accounting for 58.5% of alleles; p.Phe508del accounted for 16.7%, while 41.5% of alleles remained unidentified despite extensive screening [41]. In Libya, a small study of 10 unrelated patients found a different mutation distribution, with p.Glu1104Ter accounting for 40% of alleles and p.Phe508del for 30%, while 15% remained unidentified [42]. Moroccan data are more limited as described above. Population screening of 150 healthy adults identified p.Phe508del in two participants [31]. Because this was a carrier study of a small sample rather than a cohort of PwCF, it does not define the CFTR variant spectrum on a country level. In Egypt, a 36-variant panel genetic test has positively identified 50 of the 120 CFTR alleles in 60 children. Among the detected alleles, p.Phe508del accounted for 58% and 2183AA>G for 10% [43]. A more recent Egyptian cohort of 31 children found p.Phe508del in 35.5% of patients and reported four novel CFTR variants [44].

These differences in CFTR variants have practical implications for disease diagnosis in the region. Variant panels that are developed mainly to detect variants common in European populations may provide incomplete coverage of variants in the Arab populations. This limitation supports the need for adoption of diagnostic approaches that consider the local variant distributions.

Regional CFTR variation has also important implications for phenotyping pancreatic sufficiency status and nutritional risk. Qatar has a well-described example of a locally common CFTR variant which is associated with pancreatic sufficiency. The p.Ile1234Val (I1234V) variant was described in a large Qatari Bedouin kindred and was subsequently found in a substantial proportion of PwCF in Qatar. Fecal elastase-1 testing in the originally described kindred confirmed the presence of pancreatic sufficiency in all patients studied [45]. Preserved exocrine pancreatic function, however, did not necessarily imply nutritional adequacy [23,46].

Diversity of CFTR variants has important implications that extends to eligibility for modulator therapy [13]. This is particularly relevant in the Arab world, where recognized treatment-responsive variants coexist with rare and locally enriched variants that remain incompletely characterized and may not be amenable to currently available modulators [4].

## 4. Nutritional Manifestations and Clinical Outcomes

### 4.1. Undernutrition and Growth Faltering Are Dominant

Nutrition-specific data in the Arab world come mainly from studies of pediatric cohorts in several parts of the Arab world. Despite differences in study design, definitions, and treatment era, undernutrition and growth faltering are recurring findings. Table 1 provides a summary of relevant regional studies reporting growth, anthropometrics, and nutrition-related outcomes.

In Bahrain, Isa et al. reviewed 47 children with CF and pancreatic insufficiency and found that 72% met the study definition of nutritional failure [47]. Nutritional failure remained common despite reported use of PERT and high-calorie supplementation in majority of children. Among those with nutritional failure, more than half had both wasting and stunting. Because the study included patients who were diagnosed over more than 30 years, and did not report outcomes by treatment era, the findings may not reflect current nutritional care or outcomes.

Multiple Saudi studies, on the other hand, provide data that span different periods of care. A retrospective review of 190 patients between 1993 and 2002 reported mild-to-moderate malnutrition in 65% and stunting in 63% [54]. Low weight-for-height, low serum albumin and abnormal red-cell indices at follow-up were associated with early mortality. These findings provide important historical evidence linking nutritional status with survival, although they reflect an earlier treatment era. Recent data suggest that malnutrition remains a substantial problem. In a 14-year retrospective cohort from Dammam, which included 48 children between 2010 and 2023, 60.4% were malnourished with low body mass index (BMI) z score of −1.61 [55]. Malnutrition, in this cohort, was associated with delayed diagnosis, more frequent hospitalizations, uncontrolled fat malabsorption, and poor appetite. Of note, age and poor appetite/intake remained independently associated with malnutrition in the final model.

In Egypt, El-Koofy et al. assessed 50 children before and after a three-month individualized nutritional rehabilitation program that implemented dietary counselling, vitamin and mineral supplementation, and adjustment of PERT [48]. At baseline, 78% of patients had weight-for-age z scores ≤ −2, 48% of patients had BMI z scores ≤ −2, and 62% of patients had height-for-age z scores ≤ −2. Low albumin, low prealbumin, and anemia were reported in 29%, 80%, and 43%, respectively. After the 3-month program, among the children with available follow up data, the proportion with low weight-for-age decreased from 78% to 40%, children with low BMI from 48% to 32%, children with low prealbumin from 80% to 41%, and children with anemia from 43% to 17%. Although anthropometric and biochemical measures improved, the uncontrolled design of the study and the short follow-up period limit definitive conclusions about the effect of the intervention.

More recent data from Egypt show that nutritional concerns remain evident. In a single-center registry at Ain Shams University, which included 120 children with CF followed between 2022 and 2024, failure to thrive was reported in 58.3% [25]. At the same center, a 12-month quality-improvement project followed 45 PwCF aged 6–21 years old [50]. Among the 20 patients who did not receive elexacaftor–tezacaftor–ivacaftor (ETI), the mean BMI-for-age-z score improved from −0.88 to −0.13. The mean ppFEV_1_ increased from 57.9% to 65.0%. The improvement project implemented a multidisciplinary care approach consisting of nutritional support, airway clearance, caregiver education, and telehealth follow-up. Participants who received modulator therapy during the second phase of the project experienced additional improvement.

A multicenter retrospective study from Upper Egypt reported malnutrition in 75 out of 104 (72%) children with CF, including severe malnutrition in 36 (35%) [49]. Among children who were able to perform spirometry, severe malnutrition was associated with markedly lower ppFEV_1_.

Malnutrition and poor growth were reported in pediatric cohorts elsewhere in the region. In a Palestinian cohort of 77 PwCF, mean BMI was 15.9 kg/m^2^ and all five children younger than 2 years with available data had a weight-for-length below the 5th percentile. Moreover, nutrition-related domains of a validated CF-specific quality-of-life questionnaire including weight and body image were also notably impaired [52]. In Sudan, a retrospective series of 35 children found weight < −2 SD in 31 (88%), height < −2 SD in 26 (75%), and wasting in 30 children (86%) [30]. Contemporary data from Jordan also showed poor nutritional status. In 50 PwCF, the mean BMI was 15.7 kg/m^2^ and the mean BMI z score was approximately −1.6 [51]. In a Syrian cohort of 173 children with CF, failure to thrive was reported in 61.3%, chronic diarrhea in 46.8%, and steatorrhea in 34.7%. Mortality rate was 23.1%, with 33 (82.5%) of 40 deaths occurring in the first year of life [26].

Tunisia provided one of the largest North African datasets on nutritional status. A national multicenter series of 123 children with CF reported undernutrition in 55.2% at presentation, and chronic diarrhea in 41.4% [28]. Among 116 children who had follow-up, 56% had hypotrophy, defined as weight below −2 SD, and was independently associated with mortality.

Recent Algerian data provided a similar perspective. Among 34 children with CF, 38.2% were undernourished, including 20.6% with severe malnutrition, and 29.4% had stunted growth [29]. All children were reported to have pancreatic insufficiency and were receiving treatment.

In summary, undernutrition and growth faltering remain the dominant nutritional problems reported in published pediatric CF cohorts from several Arab countries. Poor nutritional status was associated with reduced pulmonary function, greater disease severity, and mortality in several cohorts. Such relationships are considered further in the next section.

The published studies rely heavily on isolated anthropometric measures. However, contemporary CF nutrition care emphasizes serial growth trajectories together with assessment of dietary intake, body composition, pancreatic and gastrointestinal status, micronutrient and biochemical measures, physical function, and quality of life [7,8,56]. Many of these measures are rarely reported in studies from the Arab world.

### 4.2. Nutrition, Pulmonary Function, and Survival

The relationship between nutritional status and lung disease in PwCF is well established internationally, and in some regional studies [1,8]. In Upper Egypt, children with severe malnutrition had substantially lower spirometry indices than those with better nutritional status [49]. Among adults with CF in the UAE, median BMI was approximately 19 kg/m^2^ and median ppFEV_1_ was below 50%, besides the frequent *Pseudomonas aeruginosa* infection and pulmonary exacerbations [19]. Even though these studies are largely cross-sectional, the poor nutritional and pulmonary status commonly occurred together.

Several regional studies have also linked nutritional status with mortality. In a Saudi cohort of 190 patients followed between 1993 and 2002, 26 (14%) patients died. Low weight-for-height and low serum albumin at follow-up were associated with earlier mortality [54]. A Tunisian national cohort reported that among 62 children with CF who died, the median age at death was 8 months. Hypotrophy, defined as weight below −2 SD, was independently associated with mortality [28].

High mortality, particularly during infancy and childhood, has also been reported in several cohorts in Arab countries. In a historical Jordanian series of 202 cases, 38 children died, majority of them before 1 year of age [36]. In Syria, 40 of 173 children died, including 33 during the first year of life. Seven deaths were attributed to malnutrition, while pulmonary sepsis and meconium ileus accounted for most of the remainder [26]. In Oman, 53 of 227 patients followed at two referral hospitals between 2006 and 2020 had died by the end of the study period. Mortality in that study differed considerably by birth cohort: only 47.7% of those born before 2000 were alive at the end of follow-up compared with 90.8% of those born between 2010 and 2020 [22]. An earlier Bahraini study also reported a marked decline in first-year mortality across successive calendar periods in a small cohort of patients with severe growth faltering and hypoalbuminemia [57].

While not reflective of population-level data or contemporary survival estimates, the published data on mortality thus far point to a concerning regional picture.

### 4.3. Pancreatic Function, Pancreatic Enzyme Replacement Therapy, and Persistent Malabsorption

Exocrine pancreatic insufficiency (EPI) remains a major contributor to malnutrition in PwCF. However, pancreatic status and adequacy of PERT are not well characterized in much of the regional literature.

In the Bahrain cohort, all children with EPI were reportedly prescribed PERT, yet nutritional failure remained common [47]. Prescription of pancreatic enzymes alone, therefore, provides little evidence for whether EPI related malabsorption is adequately treated.

Similarly, a Saudi pediatric cohort provides further evidence that persistent malabsorption remains clinically relevant despite prescription of PERT. Uncontrolled fat malabsorption was associated with malnutrition in univariable analysis, although it was not retained in the final multivariate model [55]. In the previously cited Egyptian nutritional intervention program, adjustment of PERT based on symptoms alongside individualized dietary care and supplementation, were among the interventions associated with improved nutritional measures [48].

Regional CFTR variations are also reflected in the variation seen in the pancreatic phenotype. In an older UAE genotype-phenotype study, all 15 children who were homozygous for p.Ser549Arg were found to be pancreatic insufficient [58]. Qatar provides an important contrast where all who were homozygous for p.Ile1234Val were found to have normal fecal elastase-1 concentrations, confirming preserved exocrine pancreatic function [45]. A later report from what seems to be the same or substantially overlapping cohort in Qatar showed preserved linear growth and normal BMI [53].

Elsewhere in the region, pancreatic involvement is often described by symptoms alone rather than by use of objective measures. In the Syrian cohort, symptoms related to steatorrhea were common, but evaluation of pancreatic function was incomplete. The authors identified the need for objective assessment of pancreatic function as a priority for more reliable phenotyping [26].

Current nutrition guidelines recommend objective assessment of pancreatic function. Fecal elastase-1 is the principal non-invasive diagnostic test [7]. Persistent poor growth, steatorrhea, or other gastrointestinal symptoms should prompt review of PERT dosing, timing, administration technique, and level of adherence, as well as consideration of other potential gastrointestinal or nutritional causes, rather than automatic escalation of calorie intake or enzyme dose [59]. Pancreatic status may also warrant continued assessment over time in patients who receive CFTR modulators, particularly in younger children, as emerging evidence suggests that exocrine pancreatic function can improve [60].

In summary, the lack of details needed to evaluate pancreatic insufficiency and its treatment is an important and persistent limitation of the regional literature. Objective assessment of pancreatic function is rarely reported. Moreover, studies do not provide sufficient information on PERT dose, administration, adherence, gastrointestinal symptoms, or ongoing malabsorption. Linking objective measures of pancreatic status and PERT with longitudinal growth and nutritional outcomes should be a priority for future studies in regional cohorts and registries.

### 4.4. Micronutrients and Body Composition

Data regarding micronutrient status in PwCF in the Arab World are also limited. However, vitamin D levels have been examined in several cohorts. In Saudi Arabia, children with CF had significantly lower mean serum 25-hydroxyvitamin D concentrations than healthy controls [61]. Data from Bahrain that included a subset of a CF Center’s population, identified vitamin D deficiency in 28 of 34 children tested and vitamin E deficiency in 9 of 35 children [62].

Studies from Qatar provided information on vitamin D and bone health in 40 PwC, homozygous for p.Ile1234Val, all of whom were pancreatic sufficient, 60% had 25-hydroxyvitamin D concentrations below 20 ng/mL despite their preserved linear growth and BMI [53]. A later study from the same kindred, which likely included an overlapping group of patients, found vitamin D deficiency in 20 of 26 patients; dual-energy X-ray absorptiometry (DXA) also identified reduced bone mineral density in 7 of 26 patients [63]. These findings show that vitamin D deficiency and reduced bone mineral density can occur despite preserved exocrine pancreatic function.

Studies from Egypt also provided body-composition data. In a small case–control study of 15 children with CF, DXA showed lower fat mass, lean mass, and bone mineral measures than in healthy controls. Four children had osteopenia. Low fat-free mass was associated with lower bone mineral density [64]. The later Egyptian nutritional rehabilitation study used triceps skinfold thickness and mid-arm measurements and reported improvements in hemoglobin, albumin, and prealbumin after intervention, although these biochemical measures can also be influenced by inflammation and disease activity [48].

Beyond these small studies, regional data on micronutrient and body-composition outcomes remain sparse. Biochemical assessments of vitamin levels, iron status, levels of essential fatty acids, or other micronutrients are rarely reported. Longitudinal data on bone health and body composition are also lacking. As nutritional phenotypes change with CFTR modulator therapy, future regional studies should include measures of fat-free and fat mass, muscle function, and bone health alongside conventional anthropometry.

## 5. Delivering CF Care Across Diverse Health Systems

### 5.1. Access and Continuity of Nutritional Care

Early diagnosis of CF and timely entry into standardized multidisciplinary CF care are important for preventing growth faltering, identifying exocrine pancreatic insufficiency, and addressing nutritional complications before they become established [7,8]. The infrastructure required for newborn-screening programs exists in several Arab countries, but CF is not included in most of these countries’ screening panels [65]. Jordan and Saudi Arabia, for example, have established newborn-screening programs that do not include CF. The UAE has a rapidly expanding but not yet universal CF screening program [66]. Delayed diagnosis of CF, therefore, remains common in several regional cohorts [3,6,55].

Published reports document the presence of centers or clinics that appear to focus on CF care in some parts of the region, although information on reach and continuity of that care is limited. In the UAE, for example, almost all indigenous adults with CF were reported to be followed at a single specialized clinic [19]. Jordan registry identified that patients are followed by different healthcare sectors but highlighted incomplete standardization of care and lack of data integration [24].

In Egypt, 28 out of 133 (21%) of surveyed physicians indicated dietitian support for CF as readily available, while 66/133 (49.6%) reported that it was rarely or never available [67]. In a small regional survey of 10 pediatric CF practitioners, specialized nutrition support was reported by 2 out of 5 respondents from lower-income setting and 4 out of 5 from higher-income settings [68]. Regional information on dietitian staffing for CF care, frequency of nutritional review, access to tertiary centers, and continuity through transition to adult care is largely lacking.

Egypt provides regional examples of nutritional care as part of a structured multidisciplinary program with positive results in a three-month rehabilitation program and a more recent quality-improvement initiative at Ain Shams University. The nutritional and pulmonary outcomes of that program were discussed in Section 4.1 and Section 4.2 [48,50]. Further, in Jordan, PwCF who attended regular clinic follow up visits for more than three-years, had higher BMI and ppFEV_1_ than their counterparts with sparse follow up [51].

Use of oral nutritional supplementation was reported in several regional cohorts but was poorly characterized. In Bahrain, 89% of children received high-calorie supplements, [47] while 58.3% of the Saudi pediatric cohort received high-calorie supplementation and 5 of 48 received enteral feeding [55]. In Tunisia, intermittent gavage was reported in 3 of 32 children [27]. The Egyptian interventional protocol included use of enteral nutrition support by mouth or through nasogastric tube if needed. However, the number frequency of using tube feeding was not reported [48]. Across these studies, indications, duration, adherence, and nutritional response to these interventions were not completely described. Regional studies provide very limited evidence regarding outcomes of enteral feeding.

The decision and timing of escalation to enteral feeding may depend on clinical stability of the patient, severity of pulmonary disease, access to experienced multidisciplinary teams, and the patient and family preferences. These factors have been minimally examined in the published regional cohorts.

Early diagnosis of CF, trained dietitian care, reliable access to PERT and nutritional supplements, standardized monitoring, and close follow-up remain fundamental to improving nutritional outcomes [7,8,48,50]. CFTR modulators can substantially improve outcomes in eligible patients, but their benefits are unlikely to be fully realized when diagnosis was delayed and care was fragmented [7,8,13].

### 5.2. CFTR Modulators and Nutritional Transitions

Published data on nutritional outcomes of CFTR modulator therapy in the Arab world are sporadic and very limited. In Oman, 21 children with at least one p.Ser549Arg variant were treated with ivacaftor and had a reported increase in BMI by 1.9 kg/m^2^ after 48 weeks of treatment among those completing follow-up [69]. In Saudi Arabia, two severely undernourished siblings who were homozygous for p.Ile1234Val were reported to have marked improvement in BMI z scores after eight months of ETI [70]. At Ain Shams University in Egypt, patients who started ETI during the second phase of a multidisciplinary quality-improvement program had improvements in BMI z score and lung function, although the effects of ETI could not be separated fully from the concurrent intervention [50]. A small UAE cohort of 12 adults with uncommon CFTR variants, nine of whom had previously received ivacaftor, also showed improvement in lung function but a modest andnon-significant increase in BMI after ETI [71]. These regional reports mainly describe recovery from poor nutritional status rather than a shift toward overweight or obesity.

The diversity of CFTR variants across Arab populations has clear implications for CFTR modulator eligibility. The 18 variants considered potentially Arab-specific by Al-Sadeq et al. at the time of their review highlight the need for functional, clinical, and phenotypic studies to establish their responsiveness to therapy [4]. Using genotype data from CF registries and clinics worldwide, Burgel et al. estimated that extending ETI eligibility beyond p.Phe508del to other variants with established responsiveness could increase genotype-based eligibility from 82% to at least 91.5% of PwCF. The largest relative increases occurred in countries where p.Phe508del was less common [72].

Regional studies of modulator therapy rarely report the proportion of PwCF eligible for treatment, the percentage of eligible PwCF who received the treatment, or the differences in patient access within and between countries. Large global disparities in access to ETI have been documented [73], and reports of treated patients from individual tertiary centers cannot be interpreted as evidence of equitable access.

Beside genetic eligibility, access depends on affordability, regulatory approval, reimbursement, and reliable procurement. Even within the same country, access can differ according to residency status, insurance coverage, and reimbursement criteria [14]. The annual cost of ETI in the US has been reported in 2024 to be at US$ 322,000 per patient [73]. The formidable price represents a significant financial barrier, though does not necessarily represent negotiated prices or costs of the medication in Arab countries. In addition to cost, the absence of marketing applications, limited commercial incentives in smaller markets, and unsuccessful price negotiations can present additional barriers to availability [14]. Patent protection may also restrict competition from lower-cost generic alternatives.

A Turkish multicenter study covering 2021–2024 documented declining lung function during treatment interruptions under court-dependent access arrangements, illustrating the importance of continuity of access [74].

A lower-cost generic formulation offers alternative route to improving to ETI. In 2026, the CF Buyers’ Club, a volunteer parent group based in the UK was established to spread awareness and improve access to alternatives to expensive modulators, such as Triko^®^, a generic ETI formulation of Trikafta^®^ with a quoted annual adult medicine price of US$12,750 [75]. Regulatory requirements, importation, and continuity of supply nevertheless remain important considerations [14,73].

Genetic eligibility, insurance coverage, long-term affordability, and continuity of supply need to be considered by policymakers when planning CFTR modulator programs. Expanding modulator access should proceed alongside efforts to strengthen basic CF care and build sustainable, standardized, interdisciplinary CF care programs [14,76].

Modulators need to be delivered within a functioning CF care system. Earlier diagnosis, multidisciplinary follow-up, reliable access to PERT and nutritional support, and consistent monitoring are needed to maximize treatment benefits and identify adverse effects.

The nutritional gains reported in regional studies are encouraging, but in many patients, they follow years of established malnutrition, during which pulmonary health may already have been affected. CFTR modulator therapy therefore cannot fully substitute for timely diagnosis and effective nutritional care early in the disease course.

Initiatives from the World Health Organization (WHO) and the Middle East Cystic Fibrosis Association (MECFA) supporting patient access to treatment and strengthening CF care are discussed further in Section 6.

In summary, undernutrition and growth faltering therefore remain prominent nutritional concerns in the region as suggested by the available literature. Experience from countries with established CF care and modulator use suggests that excess weight gain, significant changes in body composition, and cardiometabolic risk may become increasingly relevant as access to ETI expands [13]. Nutritional care in the region will need to address both potential realities rather than assume a uniform shift from undernutrition to overweight.

### 5.3. Culture, Food Security, War, and Displacement

Nutritional care of children takes place in families and within local food systems. Regional CF studies report little information regarding customary diets, fasting practices, food-related beliefs, household decision-making, and availability and affordability of nutritional supplements and high-calorie foods. Dietary guidance in CF, therefore, needs to be adapted to locally available foods and family practices while preserving established nutritional standards [8,77].

Regional CF studies suggest that socioeconomic difficulties are an important concern. In Algeria, 73.5% of children in one CF cohort came from low or lower-middle socioeconomic backgrounds and 52.9% did not have social-security coverage [29]. In a recent Egyptian CF registry, half of the patient cohort were classified as having middle-level socioeconomic status [25].

Studies in the wider pediatric population also show substantial food insecurity across the region. In a study of preschool children in the north of Jordan, 58.2% lived in households with moderate or severe food insecurity [78]. Nationally representative Egyptian data linked poverty with inadequate dietary diversity among families with young children [79]. Severe household food insecurity has also been documented in Saudi Arabia [80]. Even though these studies were not CF-specific, they describe the circumstances in which families are being asked to meet the additional nutritional demands needed to care for PwCF. A Turkish CF cohort provides a useful regional comparison: 46.8% of 290 participants experienced household food insecurity, including 18% with very low food security. BMI was lower in the latter group [81]. Household food security was rarely assessed in regional CF studies.

War and population displacement can make food insecurity-related challenges considerably harder. Among 87 Arab refugee children with CF treated at 14 centers in Türkiye, 80 (92%) were Syrian refugees. Despite their access to the Turkish healthcare system, they had poorer nutritional status and greater *Pseudomonas aeruginosa* colonization than children in the Turkish national registry [16]. Inadequate dietary intake crowded living conditions, cultural differences, and language barriers were cited among possible contributors.

The devastation, destruction, and humanitarian catastrophe in Gaza can pose immense challenges to PwCF there. However, we identified no CF-specific data on the nutritional status or clinical outcomes of PwCF during the current war. Studies of the wider Gazan population during the war have documented increasing acute malnutrition among young children [82] and major disruption of healthcare for people with chronic diseases during the war [83]. Routine follow-up for chronic disease fell markedly during the war. Lack of medication was an important reason for interrupted treatment [83]. For PwCF, who depend on adequate food intake, PERT and other medications, airway clearance, and regular multidisciplinary care, disruption on this scale is particularly concerning. Its effects on growth, pulmonary health, and survival are yet to be clearly documented.

Reports of the experience after the 2023 earthquakes in Türkiye provide a useful regional comparison of what can happen to PwCF when medical infrastructure is severely disrupted. Among 255 PwCF in the affected provinces, 53.3% experienced food insecurity, 34.5% had disruption of routine visits, and 20.8% reported disruption of their daily CF care [84].

For PwCF living through war and forced displacement, potentially helpful strategies include utilization of patient-held medical records, mobile or outreach care teams when feasible, locally adaptable nutrition guidance, and mechanisms to prioritize and maintain access to essential medications such as PERT [85,86].

## 6. Data Gaps, Registries, and a Regional Research Agenda

The published data on CF-related nutrition in the Arab world remains fragmented. Studies use different definitions, anthropometric measures, and outcome metrics to assess nutritional status, limiting comparability across cohorts. Standardizing anthropometric assessment and reporting using consistent reference standards is an important first step toward improving longitudinal follow-up and understanding regional nutritional outcomes. A shared core data set would also improve consistency of the data collected and can be very helpful in research, clinical care, and public health policies. At a minimum, this core data should include survival status, age, timing and method of diagnosis (sweat chloride, newborn screening), genotype, pancreatic function, PERT use and adherence, anthropometry and growth trajectory, dietary assessment, oral or enteral support, micronutrient status, pulmonary function, liver disease and other system complications, and CFTR modulator eligibility and use. Food security, financial barriers, and access to treatment also deserve attention. Documentation of instability or interruptions in care should also be considered when PwCF are affected by displacement or humanitarian emergencies.

The Jordanian electronic registry shows that national data collection is possible in the region. However, missing important information such as genotype, spirometry, and anthropometric measures limits its usefulness [24]. Building a useful registry requires more than agreeing on which variables to collect. Standard definitions, trained staff, routine quality checks, appropriate governance, and sustained resources are essential. Registries can serve not only as sources of epidemiologic data, but also as tools for improving clinical care by identifying areas of priority, gaps in care and knowledge, and for comparing outcomes over time and between centers. They also give important feedback into clinical practice and public-health decision making.

A single Arab registry is unlikely to be feasible. Alternatively, linking national and center-based registries through a shared core dataset, on the other hand, may offer an alternative and more practical approach. Reports from centers in the UAE, Oman and Qatar already show the value of systematic data collection [19,21,23]. The pan-Arab genotype review provides a reference point for systematic genetic reporting [4]. The European Cystic Fibrosis Society Patient Registry (ECFSPR) Partnership Project, which supports development of harmonized registries low- and middle-income countries outside Europe, provides a practical model for shared data standards, training, quality control, and reporting [87].

International and regional initiatives can support both treatment access and the development of CF services. In 2025, WHO added ETI to its Model Lists of Essential Medicines for adults and children. The WHO Expert Committee identified generic competition and pooled procurement as potential approaches to improving affordability [88].

At the regional level, the Middle East Cystic Fibrosis Association (MECFA) has supported the provision of sweat chloride testing equipment and training for CF teams [14]. Such partnerships could help centers with limited resources establish reliable diagnostic services, train staff in standardized nutritional assessment, and develop longitudinal data collection.

Multicenter studies within each country as well as multinational collaborative studies can foster quality data generation, standardization of data collection and improved overall care.

Future research should focus on the most impactful clinical questions that remain unanswered. Prospective multicenter studies are needed to evaluate common interventional strategies that prevent poor nutrition and promote growth in PwCF. Studies on EPI in the Arab world should report objective assessment of pancreatic function and provide detailed information on PERT use as discussed in Section 4.3. Studies evaluating micronutrient status should report supplementation used, pancreatic status, dietary intake, and the presence of concurrent inflammation or infection. With the expanding use of modulator therapy in the region, studies evaluating their effectiveness should document baseline nutritional status, dietary intake, body composition, and metabolic outcomes. Genetic studies should link CFTR variants with pancreatic function, nutritional trajectory, pulmonary outcomes, and treatment response. For rare and unique variants, functional studies will also be important if clinical significance or potential responsiveness of these variants to therapy remains uncertain [4].

## 7. Discussion

**Figure 1:**
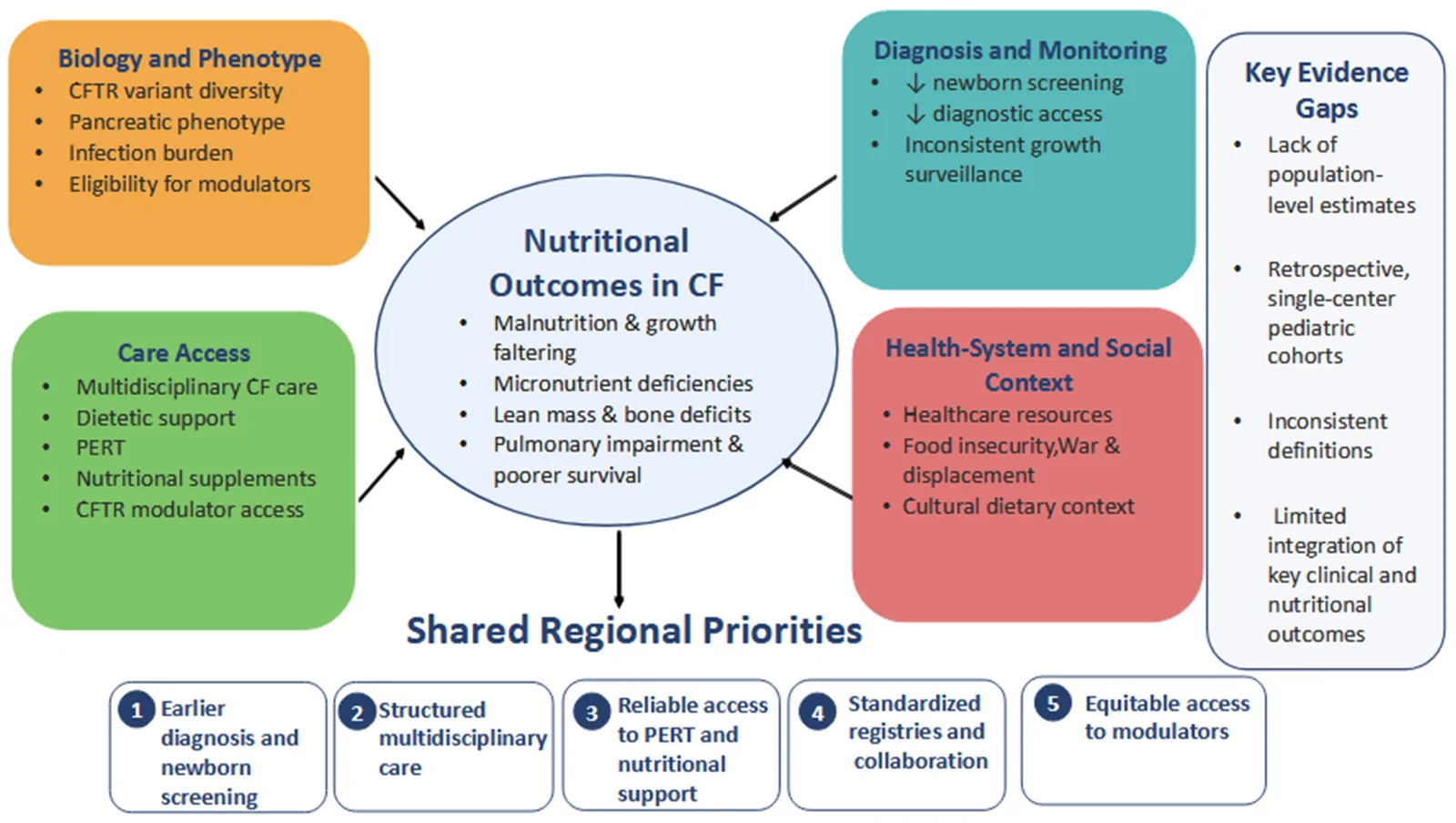
Conceptual framework for determinants, evidence gaps, and shared regional priorities shaping nutritional outcomes in cystic fibrosis across the Arab world.

This review shows that undernutrition and growth faltering remain the main nutritional problems in published CF cohorts from several Arab countries, patterns vary considerably. These variations reflect differences in CFTR genotype, pancreatic function, age at diagnosis, pulmonary disease, access to PERT and nutritional support, multidisciplinary care, eligibility for and access to CFTR modulators, and the social conditions in which treatment is delivered. Figure 1 shows a framework for these interacting variables, shared regional priorities, and important evidence gaps.

Poor growth and undernutrition recur in both older and more recent pediatric cohorts across several parts of the region [25,28,47,49,51,55]. Poor nutritional status also frequently occurs alongside more severe disease. Despite their limitations, conclusions from the reviewed studies support the importance of nutrition monitoring with early recognition and treatment of faltering.

The regional literature is uneven in both volume and type. Some countries are represented by relatively large cohorts or registries, while others appear mainly in genetic studies or small series. Published evidence therefore should not be taken as a direct measure of disease burden or quality of care, and limited clinical reporting cannot establish that CF is either uncommon or associated with worse outcomes in these settings. Morocco illustrates this gap: model-based carrier data suggested a possible burden of CF that is not reflected in the published clinical literature [31].

Biological and genetic factors also play an important role in shaping nutritional outcomes. The Arab world includes considerable ethnic and ancestral diversity within as well as between countries, and CFTR variant distributions differ accordingly [4,32]. This matters nutritionally because genotype can influence pancreatic function and eligibility for CFTR modulators. The contrast between the pancreatic-insufficient phenotype reported in the UAE and preserved pancreatic function in Qatar is particularly informative [45,58]. Differences can also occur within the same country, as seen in the distinct predominant CFTR variants reported within the UAE [35].

Studies using measures beyond anthropometry identified additional nutritional concerns. In Qatar, vitamin D deficiency and reduced bone mineral density were reported despite preserved exocrine pancreatic function [53,63], while an Egyptian study found lower lean mass and bone mineral density than healthy controls [64]. Objective pancreatic assessment, detailed information on PERT use, dietary intake, micronutrient status, body composition, muscle function, and bone health are all reported inconsistently.

Differences in nutritional and clinical outcomes may also reflect variation in healthcare systems. Many of the care gaps identified more than a decade ago, including delayed diagnosis, malnutrition, and uneven access to specialized care, remain relevant [15]. In Jordan, patients attending a CF clinic regularly for more than three years had higher BMI and ppFEV_1_ than those with sporadic follow-up [51]. Although this association cannot establish causality, it provides regional evidence for the importance of continuity of care. Further support comes from two recent quality-improvement projects at Ain Shams and Marmara Universities reporting improvements in nutritional and clinical outcomes [17,50].

Nutritional outcomes are also shaped by social determinants of health such as socioeconomic status [29], food insecurity [78,79,80], and war, displacement, and humanitarian emergencies [16].

CFTR modulators are beginning to influence nutritional outcomes in the region, although published experience remains limited. Nutritional improvement has been reported in Oman [69], Saudi Arabia [70], and Egypt [50], while a small UAE cohort showed a modest, non-significant increase in BMI [71]. These reports describe nutritional recovery from poor baseline status rather than the shift toward overweight and obesity increasingly reported in Western populations [13]. Important questions remain about equitable access to CFTR modulators [73] and the eligibility and treatment responsiveness of rare or local CFTR variants [4,73].

Regional registries can help answer many of the questions raised by this review. They should link genotype and pancreatic status with PERT use, modulator eligibility and treatment, longitudinal nutritional outcomes, pulmonary function, and survival status, while also capturing major barriers to treatment and continuity of care. Registries can also identify gaps in care and track changes in outcomes over time [89].The European Cystic Fibrosis Society Patient Registry Partnership Project provides an emerging model for data collection, training, and quality control in settings where registry infrastructure is still developing [87].

Improving nutritional outcomes across the region will require earlier diagnosis, reliable access to PERT and nutritional support, and sustained multidisciplinary care responsive to individual and social needs. As CFTR modulators become more widely available, these foundations will become even more important. The next phase of CF care in the Arab world should pair newer therapies with stronger care systems and better clinical and nutritional outcome tracking.

### Limitations

This review has several limitations. The search was narrative rather than systematic and limited to indexed English-language publications, so relevant local-language or non-indexed work may have been missed. No formal risk-of-bias or quality-assessment tool was applied because the regional evidence was limited and highly heterogenous in study design, population, and reporting. However, methodological limitations were considered during interpretation. Most studies come from pediatric tertiary centers, with relatively few adult, prospective, longitudinal, or interventional cohorts. Definitions of malnutrition and other outcomes vary, anthropometric measures and reporting are not standardized, studies span different treatment eras, and some reports from the same centers or kindreds may include overlapping patients. Information on healthcare access, nutritional services, and use of CFTR modulators is also often incomplete. These differences, together with referral bias and variable case ascertainment, preclude direct comparisons and pooled regional estimates and limit the extent to which conclusions about nutritional status and outcomes can be extrapolated to larger populations.

Despite these limitations, bringing the available evidence together provides a useful framework for understanding this complex and heterogenous landscape and helps identify which nutritional concerns have persisted, where the picture is beginning to change, and what important gaps remain.

## 8. Conclusions

Nutritional outcomes in cystic fibrosis across the Arab world reflect differences in genotype, pancreatic function, age at diagnosis, access to treatment, and the care available in different settings. Undernutrition and growth faltering remain common themes in the published literature, while the nutritional effects of CFTR modulators are only beginning to emerge. Important gaps remain, particularly in longitudinal data linking nutritional status with pancreatic phenotype, lung disease, treatment, and access to care.

Improving outcomes goes beyond access to newer therapies. Earlier diagnosis, including efforts to establish universal newborn screening, dependable access to PERT and nutritional support, sustained multidisciplinary care, and stronger regional registries remain essential.

## Figures and Tables

**Table 1:** Selected studies reporting nutritional and clinical outcomes in cystic fibrosis across Arab countries.

Study (Ref.)	Country	Design and Population	Key Nutritional and Clinical Findings
Bendoukha et al., 2024 [29]	Algeria	Retrospective; 34 children with CF (2019–2020)	Nutritional status assessed using BMI & Waterlow index; underweight and stunted growth defined as z-score < −2. Moderate and severe malnutrition reported in 17.6% and 20.6%, respectively, severity cutoffs were not reported.
Isa et al., 2016 [47]	Bahrain	Retrospective; 47 children with CF & pancreatic insufficiency (1984–2015)	Nutritional failure defined as weight-for-age or height-for-age < 5th percentile, or BMI < 10th percentile. Wasting defined as weight-for-age < 5th percentile and stunting as height-for-age < 5th percentile. Nutritional failure occurred in 34/47 (72%); among these, 19/34 (56%) had both wasting and stunting, 8/34 (23.5%) wasting only, and 7/34 (20.5%) stunting only. Low birth weight and GERD were associated with malnutrition
El-Koofy et al., 2020 [48]	Egypt	Single-center pre-/post-intervention; 50 children, 3-month nutritional rehabilitation (2016–2017)	At baseline, 60% had severe malnutrition and 38% moderate malnutrition by subjective global assessment (SGA); specific SGA scoring cutoffs not reported. Height/length, weight, and BMI z-scores ≤ −2 were present in 62%, 78%, and 48%, respectively. After 3 months of nutritional rehabilitation, these proportions decreased to 45%, 40%, and 32% among participants with available follow-up data.
Ali et al., 2025 [25]	Egypt (Ain Shams)	Single-center retrospective registry; 120 pediatric patients, 2022–2024 ^†^	Failure to thrive reported in 58.3%. The study did not report a specific anthropometric cutoff defining this category.
Saad et al., 2025 [49]	Egypt (Upper Egypt)	Retrospective multicenter study; 104 children with CF (2014–2024)	Malnutrition classified by BMI-for-age z-score: mild, >−2 to ≤−1; moderate, >−3 to ≤−2; and severe, ≤−3. Malnutrition was present in 75/104 (72.1%), including severe malnutrition in 36/104 (34.6%). Among children who completed spirometry, malnutrition was associated with lower pulmonary function; BMI z-score was positively correlated with ppFEV_1_
Hussein et al., 2026 [50]	Egypt (Ain Shams)	12-month multidisciplinary quality-improvement project; 45 PwCF aged 6–21 (2024–2025) ^†^	BMI-for-age z-score assessed using WHO standards; no malnutrition cutoff specified. Among 20 non-ETI participants, mean BMI z-score improved from −0.88 to −0.13 and ppFEV_1_ from 57.9% to 65.0% over 12 months. Additional improvement occurred after ETI initiation; bundled, uncontrolled QI intervention
Kilani et al., 2025 [51]	Jordan	Retrospective chart review, 50 PwCF (45 ≤18 years, 5 adults), single center (2016–2024)	Mean BMI 15.7 ± 3.5 kg/m^2^ and mean BMI z-score −1.6; no malnutrition cutoff specified. Regular clinic attendance for >3 years was associated with higher BMI and ppFEV_1_ than sporadic attendance.
Salah et al. [52]	Palestine	Cross-sectional, single center, 77 PwCF, (mean age 10.7 years, range 0.5–36 years) (2017)	Mean BMI was 15.9 kg/m^2^ among 72 participants; all five children aged <2 years had weight-for-length < 5th percentile. Mean age at diagnosis was 4.2 years.
Wahab et al. [53]	Qatar	Observational, single center, 40 PWCF, homozygous for p.Ile1234Val, mean age 11.9 ± 6 years (study period not reported)	Preserved linear growth (mean height Z score −0.46 ± 1.0) and BMI (20.8 ± 5.8); all had normal stool elastase; 60% had 25OHD < 20 ng/mL; height Z score correlated with ppFEV_1_ (r = 0.30, *p* < 0.05).
Banjar, 2003 [54]	Saudi Arabia	Retrospective chart review; 190 PwCF (Pediatric study, but exact ages not reported) (1993–2002)	65% had calculated weight-for-height < 90% and were classified as having malnutrition/underweight; 63% had stunted growth. Nutritional categories were based on percentage of ideal weight-for-height (severe <75%, moderate 75–79%, mild 80–84%, underweight 85–89%) and height-for-age (severe <85%, moderate 85–89%, mild 90–94%). Lower calculated weight-for-height was associated with earlier mortality.
Albrahim et al., 2025 [55]	Saudi Arabia (Dammam)	14-year retrospective single center; 48 children with CF (2010–2023)	Malnutrition defined as BMI-for-age/weight for length z-score < −1 (mild −1 to −1.99; moderate −2 to −2.99; severe ≤−3). Malnutrition occurred in 60.4% and stunting (height for age Z score < −2) in 41.7%; older age and poorer appetite/intake were independently associated with malnutrition.
Ibrahim et al., 2014 [30]	Sudan	Retrospective; 35 children with CF (Study period not reported)	High consanguinity, failure to thrive, and weight below −2 SD in 88%, height below −2 SD 75%, and wasting in 86% (no definition)
Al-Baba & Zetoune, 2021 [26]	Syria	Single-center retrospective study; 173 children with CF (2007–2017)	Failure to thrive reported in 61.3% (definition not reported); chronic diarrhea 46.8% and steatorrhea 34.7%; mortality 23.1%.
Hamouda et al., 2020 [28]	Tunisia	National multicenter; 123 children with CF (1996–2015)	Denutrition was reported in 55.2% at diagnosis (definition not reported). During follow-up, hypotrophy, defined as weight < −2 SD, was present in 56% and was independently associated with mortality.

^†^ May contain overlapping cohorts.

## Data Availability

No new data were created or analyzed in this study. Data sharing is not applicable to this article.

## Abbreviations

BMI, body mass index; CF, cystic fibrosis; ETI, elexacaftor–tezacaftor–ivacaftor FEV1, forced expiratory volume in 1 s; PERT, pancreatic enzyme replacement therapy; ppFEV1, percent predicted FEV1; SD, standard deviation.
